# Understanding and treating paediatric hearing impairment

**DOI:** 10.1016/j.ebiom.2020.103171

**Published:** 2021-01-07

**Authors:** Christian Wrobel, Maria-Patapia Zafeiriou, Tobias Moser

**Affiliations:** a Department of Otolaryngology and InnerEarLab, University Medical Center Göttingen, 37099 Göttingen, Germany; b Multiscale Bioimaging Cluster of Excellence (MBExC), University of Göttingen, Germany; c Institute of Pharmacology and Toxicology, University Medical Center, 37075 Göttingen, Germany; d Institute for Auditory Neuroscience and InnerEarLab, University Medical Center Göttingen, 37099 Göttingen, Germany

**Keywords:** Cochlea, Hearing aid, Cochlear implant, Gene therapy, Optogenetics, Stem cell

## Abstract

Sensorineural hearing impairment is the most frequent form of hearing impairment affecting 1–2 in 1000 newborns and another 1 in 1000 adolescents. More than 50% of congenital hearing impairment is of genetic origin and some forms of monogenic deafness are likely targets for future gene therapy. Good progress has been made in clinical phenotyping, genetic diagnostics, and counselling. Disease modelling, e.g. in transgenic mice, has helped elucidate disease mechanisms underlying genetic hearing impairment and informed clinical phenotyping in recent years. Clinical management of paediatric hearing impairment involves hearing aids, cochlear or brainstem implants, signal-to-noise improvement in educational settings, speech therapy, and sign language. Cochlear implants, for example, have much improved the situation of profoundly hearing impaired and deaf children. Nonetheless there remains a major unmet clinical need for improving hearing restoration. Preclinical studies promise that we will witness clinical trials on gene therapy and a next generation of cochlear implants during the coming decade. Moreover, progress in generating sensory hair cells and neurons from stem cells spurs disease modelling, drug screening, and regenerative approaches. This review briefly summarizes the pathophysiology of paediatric hearing impairment and provides an update on the current preclinical development of innovative approaches toward improved hearing restoration.

## 1 Introduction

Appropriate sensory input and normal sensory processing are required for full establishment of neural function. For example, normal hearing is required for acquisition of vocal speech and normal neurocognitive development [1]. However, hearing impairment (HI) is the most frequent sensory deficit. Disabling HI, i.e. increase in hearing threshold of greater than 30 dB in the better hearing ear, affects 34 million children worldwide [2]. Conventional hearing aids, microsurgery of the ear, also in combination with implantable hearing aids, typically achieves very good outcomes in cases of conductive HI, since they remedy the underlying acoustic attenuation. Unfortunately, we still lack causative treatment for sensorineural HI, which afflicts most HI children and results from alterations of auditory processing in the cochlea and/or the auditory nerve. Rehabilitation of mild (elevation of pure tone average threshold by 20 – 40 dB Hearing Level) to moderate (41 – 70 dB) sensorineural HI primarily builds on hearing aids. If sound amplification by hearing aids does not enable sufficient hearing, cochlear implantation is currently preferred. Criteria for implantation tend to become increasingly broad: even with hearing threshold elevation lower than 70 dB but poor aided speech understanding as well as with good low-frequency but loss of mid-high frequency hearing, children are considered to benefit from a cochlear implant and from a hybrid electro-acoustic device, respectively [3]. Clearly, novel approaches should first be demonstrated to have a favourable risk-benefit ratio in HI adults and will likely first serve children with profound HI or deafness.

**Fig. 1:**
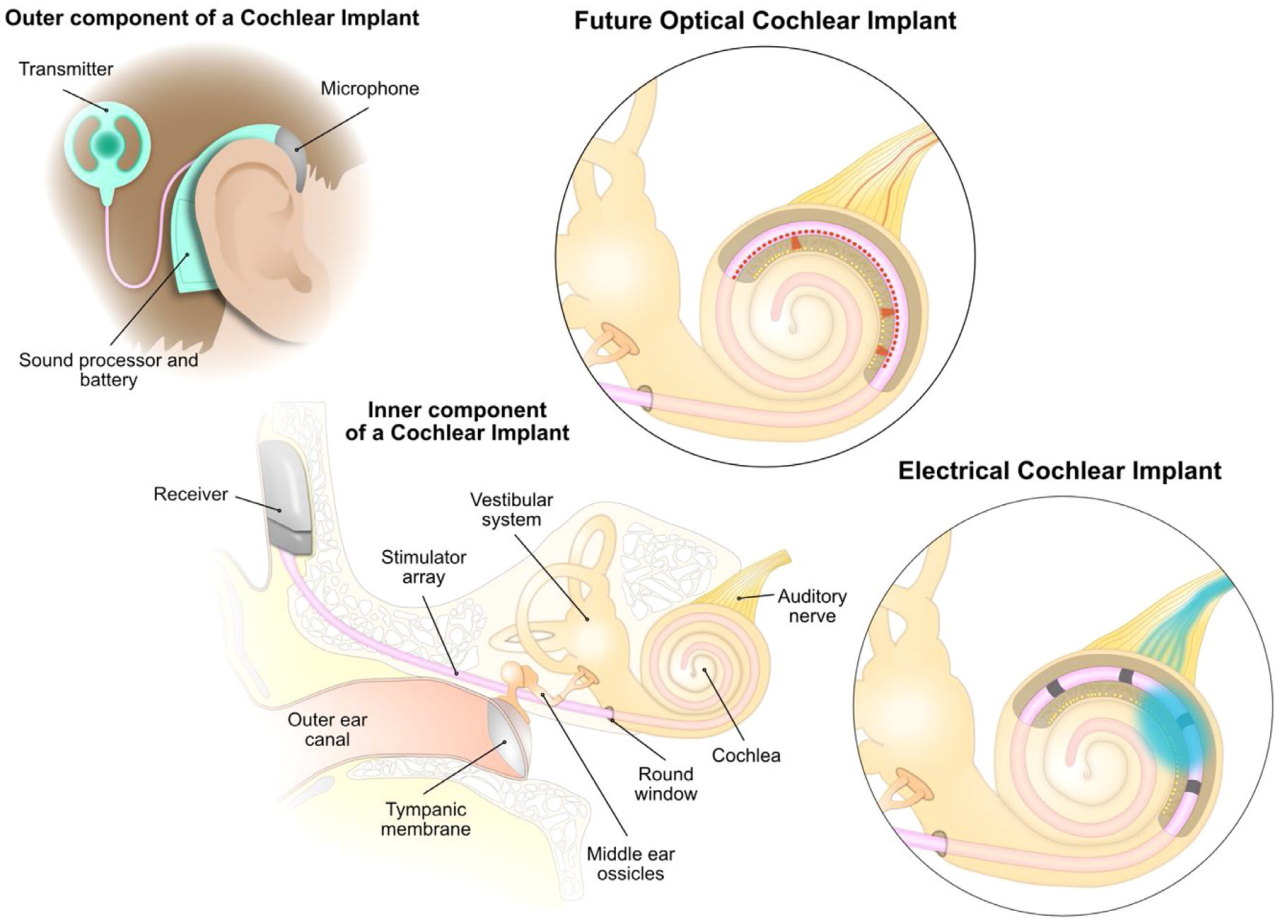
Restoration of hearing by cochlear implants: current state of care and perspectives. In cases of profound HI or deafness hearing can be partially restored by cochlear implants (CI). (Upper Left) Schematic of the external CI component: The part containing microphone, sound processor, and battery, worn behind the ear, is connected to an external induction coil magnetically held in place over the receiving coil of the internal component. (Lower left) Schematic of human ear with CI inserted (external component not drawn). From the stimulator (grey) emanates the array (pink) of electrodes in case of the current electrical cochlear implant (lower right) and emitters in future optical cochlear implant (upper right).

The electrical CI, put simply, is a medical device that encodes sound by directly stimulating the auditory nerve and thereby bypassing dysfunctional or lost hair cells. CIs consist of an external and an implanted component (Fig. 1). The external component captures and processes the sound and communicates with the implanted component via an inductive link. The implant contains an array of 12 – 24 electrodes (depending on the model and company) which is inserted into the scala tympani, a fluid-filled compartment of the cochlea. The topographical organization of the cochlea from high frequencies, activating the basal parts, to low frequencies, activating the apical parts – the so-called tonotopy – enables the perception of different pitches by stimulating distinct CI electrodes. Since the introduction of universal newborn hearing screening in many countries, the time to detection, confirmation diagnostics, and onset of rehabilitation of hearing has been shortened substantially [4,5]. Given early implantation (first year of life for congenitally deaf children) and appropriate speech therapy, the CI typically supports a nearly normal development of speech, and therefore opens up social interaction in the hearing world with possibilities of regular education and profession [6]. Generally, in cases of profound bilateral hearing impairment or deafness, the sooner diagnosis and (bilateral) implantation proceed, the better the hearing, speech, and mental development outcome [7], [8], [9], [10]. The outcome of CI rehabilitation also depends on the number of preserved SGNs, which can be reduced in neurodevelopmental, toxic-metabolic, mitochondrial, autoimmune and genetic disorders as well as due to ischaemia, neoplasm, and infections and generally declines during prolonged sensorineural HI. The neural status of the cochlea should therefore be considered when evaluating the indication for CI [20], [21], [22]. The major bottleneck restricting the efficiency of electrical CIs is the number of spiral ganglion neurons (SGNs) recruited by a single active electrode, which is rather large due to an undirected sprawling electric current in the conductive fluid of the cochlea (Fig. 1) [11,12]. The resulting channel overlap results in poor spectral resolution of sound encoding by electrical CIs, with typically less than ten distinguishable stimulation channels, primarily leading to poor speech perception in noisy environments, reduced benefits in tonal language speaking patients, very restricted transfer of voice inflections and emotions, as well as strongly limited perception of music [13], [14], [15]. Efforts are being undertaken to bring electrodes and SGNs closer together for reducing the current thresholds and potentially increasing the number of separate channels, e.g. by penetrating the auditory nerve with a multielectrode array for direct contact of electrodes and nerve [16,17]. Another approach is to attract SGN neurites to nearby electrode-arrays using neurotrophic factors (e. g. BDNF and NT3) which can be combined with further functionalization of the electrodes for neurite guidance [18,19].

Recently, optical stimulation has been demonstrated to fundamentally improve spectral selectivity, as light can be better confined in space (Fig. 1 and text below). Major efforts to improve hearing rehabilitation of children with hearing aids and cochlear implants are well justified by the fact that we do not yet have other efficient means of restoring hearing and these devices represent a “one suits all” solution, as long as there is a functional auditory nerve. Nonetheless, the genetic analysis of HI, the subsequent analysis of gene function and dysfunction in genetically tractable animals, as well as preclinical gene therapy efforts have achieved major progress, which enables well-informed counselling and promises causative treatment of select forms of hereditary HI. Sequencing a gene panel of known deafness genes is an efficient approach to uncover the likely disease-causing mutation. If this procedure is not successful, whole exome sequencing (WES) in the affected individual (and potentially the parents: “trio-WES”) or whole genome sequencing (WGS) can be performed. Genetic analysis, combined with in-depth clinical phenotyping ideally results in early definitive diagnosing and intervention with improved outcomes in hearing rehabilitation and development of the affected children (see above) and their siblings, for whom then diagnostic and appropriate intervention can be stream-lined. Aside from the informed choice of hearing aids or CI, counselling of families might include advice on protective measures. For example, when diagnosed with Pendred syndrome that results from mutation of the *SLC26A4* gene, children should avoid head- (e.g. due to contact sports) and baro (e.g. due to scuba diving)-trauma and wear head protection for activities with high risk of falling. The hypothesis for the progression of HI due to head trauma is the pressure propagation from the cerebrospinal fluid space into the cochlea via an enlarged vestibular aqueduct that is typically found in Pendred syndrome.

## 2 Disease mechanisms

**Fig. 2:**
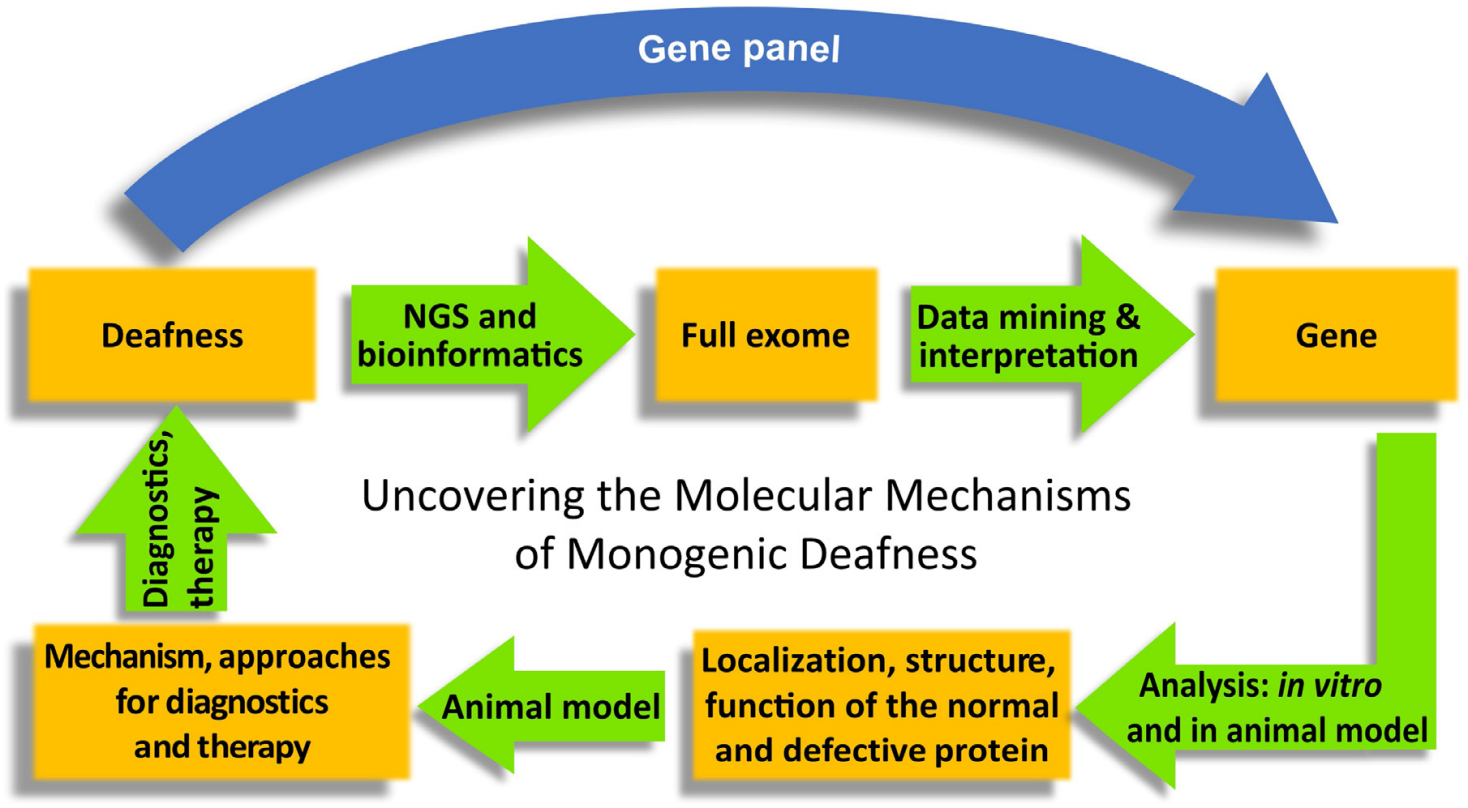
Elucidation of the molecular mechanism in cases of monogenic hearing loss. If hereditary hearing loss is suspected, sequencing a gene panel of known deafness genes is employed to uncover the likely disease-causing mutation. If this procedure is not successful, whole exome sequencing in the affected individual (and potentially the parents) will be performed. In each case, data interpretation by a Mutation Mining Team allows identification of the affected gene in the majority of cases. In vitro analysis and generation of animal models for newfound mutations may then identify localization, structure, and operation not only of the normal but also the defective protein. This allows further assessment of the disease mechanism and the development of diagnostic and therapeutic approaches that may then be applied for treatment of hearing-impaired patients.

Currently, more than 150 non-syndromic gene loci have been identified in humans [23], [24], [25]. Nowadays, application of high throughput sequencing procedures [3, 4] (next generation sequencing, NGS) has become a standard procedure in many countries (Fig. 2). In contrast to the formerly applied Sanger sequencing that typically limited the search to mutations in few genes (such as for example the GJB2 gene coding for connexin 26), NGS-based analysis (gene panels, WES and WGS) can now be performed. The decision which type of molecular diagnostics can be performed and how many deafness genes are sequenced is mainly determined by the context of the examination (scientific evaluation or routinely performed diagnostics) and the availability in a given country. In conjunction with clinical phenotyping and pedigree analysis, NGS-based sequencing then also identifies the pattern of inheritance; e. g. autosomal recessive (gene/disease is marked with DFNB, both parents are carriers of the mutation but usually healthy), autosomal dominant (DFNA, depending on penetrance one of the parents may be hearing impaired), and X-chromosomal (DFNX, the mother is carrier of the mutation but usually healthy, male descendants are affected) or identify *de novo* mutations. The sequences generated by NGS-based methods (so-called “reads”) require a careful bioinformatic analysis ideally by a Mutation Mining Team of geneticists and then allows identification of the affected gene in the majority of cases. Although large WES and WGS data sets require the expertise of bio-informaticians, it is an unbiased high throughput method to identify *de novo* mutations. This holistic approach requires the close collaboration of specialists from different disciplines as medicine, genetics and biology but is an essential step towards personalized medicine.

In-depth clinical phenotyping starts with otolaryngological and often also neurological examination, physiological and behavioural audiometric measurements of hearing and involves further disciplines as indicated by the results of genetic and clinical analysis. This typically allows to distinguish syndromic and non-syndromic disorders. Moreover, such analysis may already reveal specific pathophysiologies such as auditory synaptopathy or neuropathy with consequences for hearing rehabilitation [21,26,27].

*In vitro* analysis and generation of appropriate animal models of human hereditary HI allow the identification of localization, structure, and operation not only of the normal but also the defective protein. Animal models such as knock-out and knock–in mice, which provide access to molecular, cellular, and systems level analysis and preclinical testing of therapeutic strategies (Fig. 2). Eventually, these efforts pave the way for improved clinical diagnostics and for establishing causative treatment. As examples for such an informative analysis of human hereditary HI, we name HI caused by mutations in the *TMC1* gene [28] in DFNA36, DFNB7, and DFNB11, coding for the putative hair cell mechanotransducer channel, as well as mutations in the *OTOF* gene in DFNB9 [29], coding for the hair cell synaptic protein otoferlin. Immunolocalization places TMC1 to the tips of the hair cell stereocilia [30] and otoferlin to the basolateral compartment [31], as would be expected for proteins involved in mechanotransduction and synaptic transmission. Mouse mutants have served the analysis of *Tmc1* and *Otof* gene function and rescue by virus-mediated expression of the wild-type coding sequence has lent further support to the hypothesized gene function and paved the way for future gene therapy (see below). This way, the requirement of TMC1 for mechanotransducer currents [32] and the roles of otoferlin in Ca^2+^ triggered fusion of synaptic vesicles and their replenishment [e.g. 31,33,34] have been indicated. Moreover, this work has contributed to further establish the concept of auditory synaptopathy, a specific HI in which the synaptic sound encoding is impaired [review in ref. 26]. Besides genetic causes, congenital infections as cytomegalovirus (cCMV) account for a large amount of children suffering from bilateral sensorineural hearing loss (15–20%) [35]. Infections of a neonatal mouse model with moderate amounts of CMV identified SGN loss underlying hearing impairment [36].

### 3.1 Future gene therapy

Genetic diagnostics and preclinical work in animal models promise that gene therapy of human monogenic HI will become available within the coming decade [for a more detailed review see ref., [37]]. Given the large number of affected genes, therapies will likely become available only for a selection of monogenic HI with comparably small populations. Selection of monogenic HI for gene therapy also needs to consider the degenerative state of the cochlea, which, in some cases, is likely already profound *in utero*, prohibiting functional restoration. Gene therapeutic approaches target hair cells, SGNs, or other cellular population such as supporting cells. While gene replacement, supplementation, or correction approaches aim to restore normal gene expression, other techniques aim at transgenic strategies e.g. for expression of opsins for optogenetic hearing restoration (below) or for trans-differentiation of supporting cells to generate hair cells [38]. The latter approach has been taken to a first-in-man trial in adults employing adenovirus [ClinicalTrials.gov identifier: NCT02132130]. In most preclinical gene therapy trials, instead, non-pathogenic adeno-associated viruses (AAVs) are used that are less immunogenic than adenoviruses and have therefore been applied in gene therapy of the eye, such as in the case of Luxturna, a FDA approved gene therapy. However, given the limited packaging capacity of standard AAV (<4.7 kb, [39]), delivery of the coding sequence of some large genes, such as *OTOF* (~6 kb) in DNFB9, is not straightforward. The prevalence of *OTOF-*related deafness accounts for up to 5–8% of autosomal recessive non-syndromic hearing loss cases in some Western populations [40] and hence, resides within the top five of genetic hearing disorders that require therapeutic intervention [1]. To overcome the packaging problem, a dual-AAV approach has been employed in DFNB9 mouse models, in which 5′ and 3′ fragments of the coding sequence are carried by two different AAVs where, both sequences are fused e.g. by recombination inside hair cells co-transduced by both AAVs [41,42]. Alternatively, overloading of single AAV has recently been shown to express otoferlin in hair cells and to partially restore hearing DFNB9 mouse models. While loss of gene function such as in DNFB7, 9, 11 and dominant HI with haploinsufficiency (DFNA36) are amenable to gene replacement, supplementation, and correction, dominant HI due to a dominant negative allele requires gene correction. *TMC1* has served as target for several of these approaches including genome editing by CRISPR-Cas9 and base editing [43], [44], [45], [46]. By utilizing homology directed repair (HDR), mutations can be corrected by CRISPR-Cas9 as shown for *TMC1* [45]. A first phase I and II clinical trial [BRILLIANCE, ClinicalTrials.gov Identifier: NCT03872479] [47] is planned to test safety and feasibility of Cas9 mediated repair of the most common cause of inherited childhood blindness (Leber's congenital amaurosis 10). The success of this trial will determine the future use of in vivo CRISPR-Cas9 trials. Since caspases have been shown to have undesirable off-target effects, elaborate pre-clinical tests are required to ensure no DNA damage is induced due to unspecific binding of the specific gRNA-CRISPR-Cas9. An attractive and safer alternative to genome editing, successfully used in the treatment of haploinsufficiency-related obesity [48] is CRISPRa. CRISPRa consists of an inactive form of Cas9 (dCas9) fused to one or more transcriptional activators. dCas9 guides the transcriptional complex to the promoter region of the gene of interest and enhances its transcription [49]. CRISPRa results in 2–3 fold upregulation of protein expression, which can be modulated by combination of different gRNAs [50]. Haploinsufficiency-related deafness as for example MYO7A mutations would thus be an interesting target for CRISPRa therapy. Although the size of Cas9 restricts the delivery of the protein by viral transduction and therefore its therapeutic application, several strategies have been employed to package the protein in viral particles [49]. One of the most recent developments is the identification of novel smaller caspases as the 70-kilodalton CasΦ, which despite its smaller size can efficiently edit the genome [51]. These caspases are attractive tools that enhance the therapeutic potential of CRISPR-Cas9. Finally, base editing offers greater specificity of genome editing [52] and has been applied to correct *TMC1* mutations [46].

### 3.2 Hearing restoration by next generation cochlear implants

**Fig. 3:**
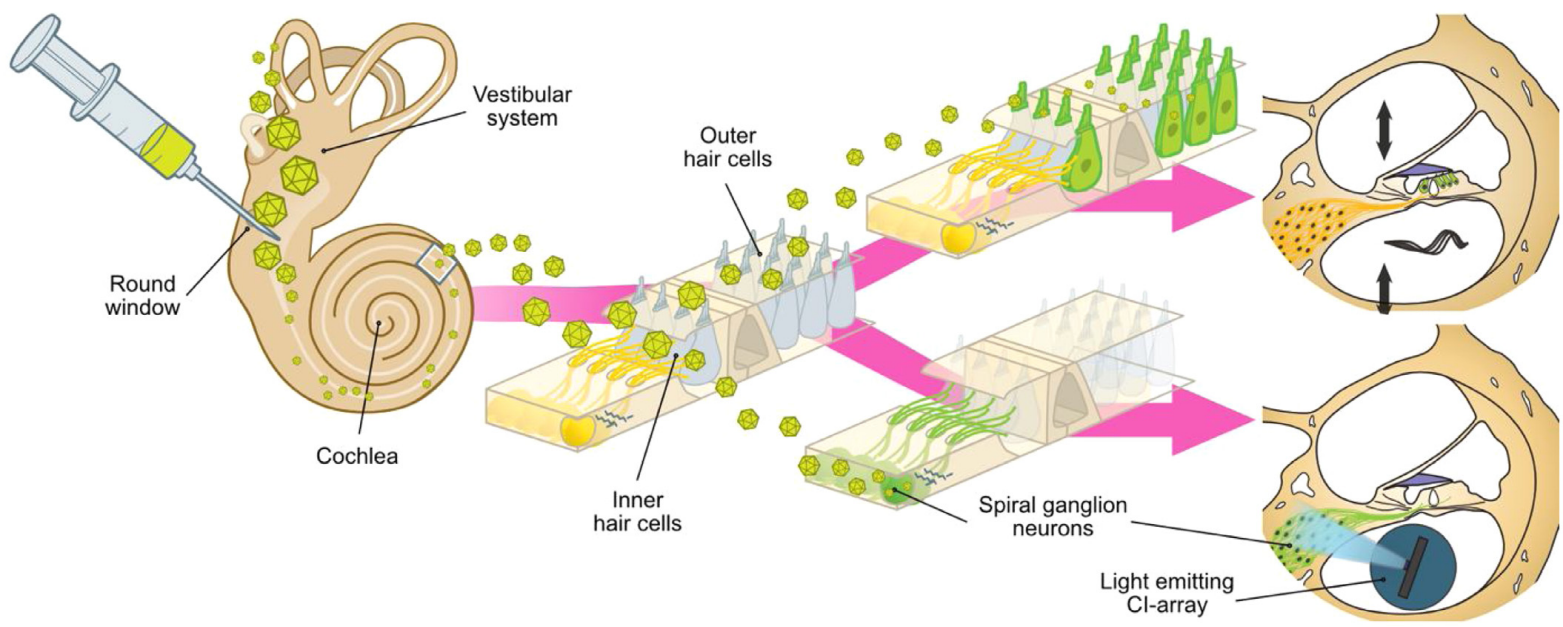
Future gene therapy of the ear. Cartoon illustrates the application of virus suspensions into the inner ear to target for gene therapeutic restoration e.g. of hair cell function (upper right) or optogenetic manipulation of SGNs for optical stimulation (lower right, axial section of a cochlear turn).

Next generation optical cochlear implants (Figs. 1 and 3) promise to overcome the aforementioned drawbacks of electrical CIs by activating much smaller populations of SGNs, since light can be better confined in space than electric current, theoretically increasing spectral selectivity to levels of normal hearing. The clinical translation of optical cochlear stimulation requires a multidisciplinary approach, comprising stable, cell-specific optogenetic manipulation to photosensitize SGNs as well as medical device engineering to develop a life-long durable light emitting cochlear implant. In a nutshell, optogenetics combines the transgenic expression of light-sensitive proteins and light application for optical control of cell functions, e.g. evoking action potentials in neurons expressing the light-gated ion channel channelhodopsin-2 (ChR2) [53,54]. Recent studies on rodents with optogenetically modified SGNs, mediated by postnatally applied AAVs, and fibre-based optical cochlear stimulation [55], [56], [57], [58], [59] went well beyond the initial proof of principle of optogenetic stimulation of the auditory pathway [60]. Intramodiolar injections of AAV2/6, carrying the calcium-translocating ChR2-variant *CatCh,* achieved an average rate of 30% *CatCh*-expressing SGNs in the adult Mongolian gerbil. This enabled optically evoked auditory brainstem responses (oABRs) – sizeable up to 200 Hz stimulation rate and comparable in amplitude and latency to the kinetics of acoustically evoked ABRs –and activity propagated to the contralateral primary auditory cortex [55]. Tonotopically aligned multi-channel recordings of neural activity in the inferior colliculus were utilized to compare the spectral selectivity of acoustic, electric, and fibre-based optogenetic (in three tonotopically distinct positions) cochlear stimulation. Activity-based analysis revealed that the spectral selectivity of optogenetic cochlear stimulation was indistinguishable from that of acoustic hearing for low and modest levels of activity and outperformed monopolar electric at all, and bipolar stimulation at medium and high intensity levels [56]. Behavioural assays using stimulus-cued avoidance tasks resulted in the establishment of single-channel optical CIs with stable oABRs more than 100 days beyond implantation. Moreover, they demonstrated a generalization of percepts evoked by optogenetic and acoustic stimulation as well as optogenetic hearing restoration in a model of ototoxic deafness [55].

Since high temporal fidelity of sound encoding is a major objective, promising new fast-switching channelrhodopsins (ChR) – *Chronos* and *f-Chrimson* – were applied in the postnatal mouse inner ear, supporting SGN firing rates of several hundred Hz and thus approaching acoustically evoked responses [57,58]. Furthermore, *Chronos* was introduced to SGNs via the more powerful vector AAV-PHP.B and additional ER-exiting and membrane-trafficking gene sequences were employed to successfully enhance its functional expression [57]. On the other hand, *f-Chrimson* offers the advantage of red-light activation , which potentially leads to a more efficient optical stimulation with lower scattering, deeper tissue penetration, and lower risk of phototoxicity in comparison to the aforementioned blue light-gated variants [58].

With regard to the development of the optical CI as a medical device, there are distinct advantages resulting from many years of experience in the application of electrical CIs. Most challenging is the development of a flexible array comprising dozens of power-efficient and narrowly focused light emitters, which are safely encapsulated to last for decades [61]. Currently, two main strategies are pursued in this context, *active* LED-based and *passive* waveguide-based optical CIs [62,63]. Recently, multichannel microscale light emitting diode (µLED)-based CIs could be successfully applied in rodent experiments, evoking tonotopically organized highly spectrally selective activity in the auditory system [59]. These optical devices were further minimized to a 350 µm wide and 15 mm long array of 144 individually addressable 50 × 50 µm measuring µLEDs providing high power output with minor temperature increase, suitable for in vivo optogenetic cochlear application [64].

### 3.3 Regenerative approaches

The first regenerative clinical gene therapy study is underway, which aims to regenerate lost hair cells via trans-differentiation of supporting cells using adenovirus-mediated forced expression of the transcription factor ATOH1 in supporting cells [ClinicalTrials.gov identifier: NCT02132130]. Apart from efforts to recover lost hair cells, major efforts have been undertaken to regenerate SGNs and the interested reader is referred to recent reviews on this topic [65], [66], [67].

Advancements in human induced pluripotent stem cell (iPSC) technology, genome editing technologies, as well as organoid development offer perspectives for future regenerative therapy. Human neural organoids are self-organized tissues generated by directed differentiation of human embryonic stem cells (ESCs) or iPSCs embedded in a matrix [recent review in ref. 68]. Brain organoids have been successfully used in disease modelling and drug screening [68]. In a recent study that established and characterized a novel brain organoid model, hallmarks of network development resembling features of the foetal brain, such as giant depolarizing potentials, GABA polarity switch, and neuronal plasticity, were observed [69]. Accumulating evidence [69], [70], [71] suggests that the complexity of the activity in brain organoids resembles that of the developing brain such that these cultures can serve studies of network function and dysfunction.

**Fig. 4:**
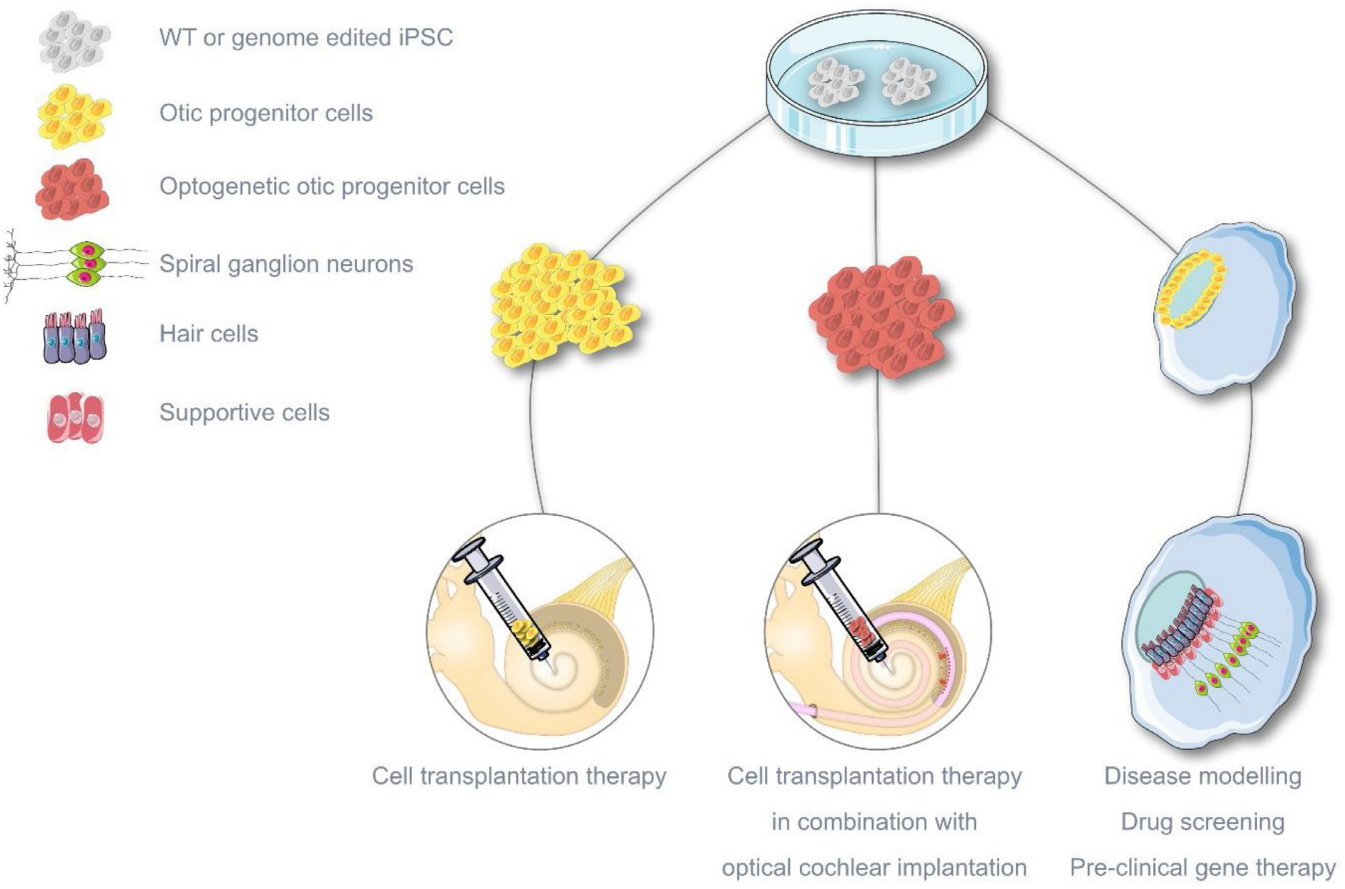
Future regenerative approaches. Scheme summarizing perspective therapeutic approaches to regenerate the inner ear and partially restore hearing. Cell transplantation (left), combination of optogenetic cell transplantation and optical cochlear implantations (middle) and otic organoid bioengineering as a potential pre-clinical model of inner ear de- and re-generation.

Similar to brain organoids, otic organoids are becoming powerful tools for disease modelling, i.e. for investigating the mechanisms of hereditary deafness. For example, in combination with genome editing, otic organoids carrying known mutations leading to genetic auditory synaptopathies could be generated for *ex vivo* studies of disease mechanisms. The same models can be used as drug screening platforms or as human preclinical models for gene therapy (Fig. 4). Along with animal models, human otic organoids can increase the translational value of pre-clinical data. Koehler et al. developed a protocol for the generation of inner ear organoids from human iPSCs [72]. Although after 2–3 months in culture only 15% of the organoids contained HC-like cells with properties of vestibular hair cells and functional mechanotransduction, this pioneering study proved that inner ear modelling from human iPSCs is feasible. Since then, a variety of protocols for generating inner ear cells from human iPSCs have been developed [for review see ref. 73] which in the future may contribute to the generation of otic organoids with higher reproducibility. Importantly, as in the brain organoid field, extensive functional tests are required in order to validate that these HC- and SGN-like cells functionally resemble the native cochlear cells. Although currently otic organoids do not resemble the unique cochlear structure, 3D bioprinting techniques such as FRESH (freeform reversible embedding of suspended hydrogels), successfully used in the cardiovascular engineering field [74], could contribute to the engineering of complex inner ear structures.

Besides disease modelling, the manipulation of developmental cues to generate human inner ear sensory cells and neurons is essential for cell replacement therapy (Fig. 4). In an ideal scenario, human HCs or SGNs would be transplanted in the cochlea to replace the degenerated cells. Unfortunately, the high sensitivity of mature HCs or SGNs to dissociation renders their transplantation technically challenging. Therefore, current preclinical transplantation experiments test the differentiation potential of otic progenitor cells (OPCs) towards all required cell populations including HCs and SGNs [75]. OPC transplantations are advantageous over the early attempts of ESC injections into the inner ear since they have a higher chance of giving rise to otic cells and lower risk for potential teratoma formations [76]. A few studies could already show that transplanted OPCs acquire similar characteristics to HCs and SGNs [76,77]. However, only one study could show partial hearing restoration after OPC injection in the denervated cochlea [75]. These data suggest that although engraftment is feasible, the injected OPC population should be defined and refined prior to injection. Combining cell transplantation with pharmacological treatments may guide OPCs to a cochlear fate (e.g. via sonic hedgehog) [78] or enhance their differentiation to hair cells (e.g. via notch inhibitors) [79]. Regeneration of SGNs by OPC transplantation in patients with major loss of SGNs, as in the case of congenital CMV infected children [36,80], could improve hearing restoration by cochlear implants. Moreover, transplantation of OPCs derived from genome-edited iPSC lines carrying channelrhodopsins such as *f-Chrimson* [58] could then be used in combination with optical CIs (Fig. 4). The safe harbour of human genome AAVS1 in chromosome 19 has been shown to allow persistent expression of transgenes in cells differentiated towards different lineages, and therefore is the ideal integration site for knock-in line generation [81].

Another important consideration for cell replacement therapy is the innate immune response. Although autologous transplantation would be from an immunological point of view the wisest option, it is expensive and time consuming. Therefore, a concerted effort from a number of labs is devoted to generating hypoimmunogenic iPSC lines. These iPSCs, edited by CRISPR-Cas9 to inactivate the highly variable HLA-I and II genes, survive allogeneic transplantation [82], [83], [84]. These technological advancements will significantly contribute to the future regenerative transplantation therapy.

### 3.4 Delivery routes and systems

Delivery of therapeutics to the inner ear is challenging as it is hidden in the dense petrous bone. Systemic application faces hurdles such as off-target effects, elimination by the immune system and low local concentrations reached due to the blood-labyrinth barrier. Smart nanoscale drug delivery vehicles targeting active transport mechanisms at the blood-labyrinth barrier could potentially overcome these limitations in the future [85]. An elegant way of administering therapeutics to the inner ear is diffusion through the round window following transtympanic injection of compound to the middle ear. Drawbacks are clearance of the compound by the Eustachian tube and restricted diffusion through the round window membrane. Efficiency of the approach can be enhanced by applying compounds in biodegradable wicks or (hydro-)gels increasing their residence time in the middle ear as well as utilizing nanocarriers, which enhance permeation through the round window membrane [86]. Intracochlear injection targeting scala tympani via the round window membrane is the most commonly used route of administration in current preclinical studies. While being more invasive than the above approaches with higher risk of further hearing deterioration, the method offers important advantages such more consistently reaching sufficient target concentrations and local action of the therapeutics [87]. Moreover, injecting AAV9-PHP.B successfully and efficiently transduced hair cells by delivery via a round window membrane approach in non-human primates [88]. Intracochlear delivery such as cochleostomy, perforating the stapes footplate, fenestration of the posterior semicircular canal (canalostomy) – possibly in combination with drug application catheters in scala tympani [87] – are more invasive with a higher risk of hearing loss. Direct administration into the cochlear modiolus has been successfully used for grafting otic neural progenitor cells [89] and AAV-mediated optogenetic manipulation of SGNs [55].

## 4 Conclusion and outlook

Universal newborn hearing screening, followed up by definitive audiological diagnostics and early fitting of hearing aids or cochlear implantation, have fundamentally improved hearing rehabilitation in prelingual HI and enabled (near) normal acquisition of vocal speech. Molecular genetics in close interplay with paediatric otolaryngology and neurology assists counselling of parents and planning of the intervention. The coming decade promises exciting new opportunities for hearing rehabilitation of select patient populations via gene therapy such as for *OTOF-*related deafness. Further down the line, optogenetic cochlear implants and regenerative approaches will likely become available, once successfully employed in adults.

## 5 Outstanding questions

Future gene therapy approaches ideally will build upon late preclinical work in non-human primate models of human monogenic HI. Safe and efficient transduction of hair cells is another important development for future gene therapy. Likewise, the optogenetic cochlear implant builds on appropriate AAV administration to the spiral ganglion that avoids spread of virus and neurodegeneration. Converting electrical to optical power, scaling up the number of stimulation channels and their simultaneous activation, as well as higher energy demands per pulse pose engineering challenges for keeping the energy budget compatible with a day-long battery lifetime. For future regenerative approaches, we need to address the nature of HCs generated from human iPSCs. Do HCs in otic organoids resemble HCs in the cochlea or the vestibular organ? And if we attain to generate cochlear HCs do they resemble inner or outer HCs? Moreover, do otic organoids develop other structural features of the inner ear as the tectorial membrane and do the otic cysts that develop contain a solution resembling the endolymph? Another important question concerning OPC implantation studies, is whether the cells injected into the cochlea and give rise to HCs, SGNs and supportive cells. And if so, do they functionally integrate with resident cells?

## 6 Search strategy and selection criteria

Data for this Review were identified by searches of Pubmed using search terms such as ear, hearing impairment, deafness, hearing aid, cochlear implant, syndrome, gene, hair cell, synapse, channel, gene therapy, virus, optogenetics, stem cells, organoids, regeneration and combinations thereof. Only articles published in English between 1983 and 2020 were included.

## Contributors

All authors performed literature search, writing and figure generation. All authors read and approved the final version of the manuscript.

## Declaration of interests

Tobias Moser is co-founder of OptoGenTech company. No conflict of interest for C.W. and M.P.Z.
